# Electrocardiography Interpretation Competency of Medical Interns: Experience from Two Ethiopian Medical Schools

**DOI:** 10.1155/2020/7695638

**Published:** 2020-05-11

**Authors:** Melaku Getachew, Temesgen Beyene, Sofia Kebede

**Affiliations:** ^1^Department of Emergency Medicine and Critical Care, Haramaya University, Harar, Ethiopia; ^2^Department of Emergency Medicine and Critical Care, Addis Ababa University, Addis Ababa, Ethiopia

## Abstract

**Background:**

Electrocardiography (ECG) is the graphical display of electrical potential differences of an electric field originating in the heart. Interpretation of ECG is a core clinical skill in the department of emergency medicine. The main aim of this survey was to assess competency of ECG interpretation among 2018 graduating class medical students in Addis Ababa University and Haramaya University. *Methodology*. A cross-sectional survey was conducted on medical interns at Addis Ababa University and Haramaya University. Data had been collected from October 01, 2018, to October 30, 2018, by using structured questionnaires. Data were entered, cleaned, edited, and analyzed by using SPSS version 25.0 statistical software. Descriptive statistics, cross-tabs, chi-squared test, Mann–Whitney *U* test, and binary logistic regression were utilized.

**Results:**

Two-hundred and two graduating medical students were involved on this survey, out of which 61.3% (95% CI 56.3–66.3%) and 32.75% (95% CI 28.25–37.25) were able to correctly interpret the primary ECG parameters and the arrest rhythm of ECG abnormalities, respectively. The ability to detect from common emergency ECG abnormalities of anterioseptal ST segment elevation myocardial infraction, atrial fibrillation, and first-degree atrioventricular block was 42.6%, 39.1%, and 32.1%, respectively.

**Conclusion:**

This survey showed graduating medical students had low competency in ECG interpretations.

## 1. Introduction

Electrocardiography (ECG) is the graphical display of electrical potential differences of an electric field starting from the heart. It is a frequently used investigation for the diagnosis of heart disease and electrolyte abnormalities. Most of the patients who visit the emergency department (ED) had ECG abnormalities. Accurate interpretation of ECG is an essential clinical skill in emergency and critical care medicine [1–3]. In day to day clinical practice, it is important for physicians and graduating medical students to know the accuracy of their ECG interpretation skills to correctly rule out the presence of cardiac disease or manage their patients [4, 5]. Even though there was no research done in Ethiopia or Africa regarding ECG interpretation skill of graduating medical students, around 9% of death in Ethiopia was caused by cardiovascular disease [6]. In Portugal, where undergraduate programs are firmly established, the overall accuracy of general practitioners (GP) for detecting ECG abnormalities were 81.0% [7]. The overall missed case rate for all seven ED was 12.8% [8, 9].

ECG interpretation skills vary among medical students, GP, and specialists. Accurate diagnosis of ECG abnormalities by a physician in any specialty contributes to appropriate clinical decision-making. Correct ECG interpretation assumes technical standards adhered to during the acquisition and recording of tracings. Many technical and patient-related factors may alter the quality of recorded ECG strips. These ECG strip artifacts in clinical practice must be recognized by the physician [1].

Appropriate ECG interpretation will improve patient care at ED and early referral to tertiary health center. However, studies from various countries have revealed deficiencies in ECG interpretation among medical students and physicians [3]. Incorrect interpretation of ECG findings can result in inappropriate management decisions with the adverse and sometimes fatal patient outcome [1, 4].

In Ethiopia, there have been no protocol in medical schools which guarantees competency in ECG interpretation. Therefore, this survey aimed to assess the fundamental skills in the interpretation of ECG among Ethiopian graduating medical students and analyzed whether the skills are developed during the process of medical education. This survey also evaluated the effectiveness of the teaching program on knowledge and practice regarding interpretation of the electrocardiogram (ECG) among medical interns of Addis Ababa University (AAU) and Haramaya University (HU), Ethiopia.

The objective of this survey was to assess competency and associated risk factors in ECG interpretation among graduating medical students or medical interns in AAU and HU.

## 2. Methodology

### 2.1. Study Area and Study Population

The survey was conducted at AAU, College of health science and HU, college of health and medical science. AAU school of medicine had emergency medicine rotation as curriculum in 4th year. HU School of medicine did not have emergency medicine rotation as curriculum. There was no emergency medicine physician in HU. The study population was AAU and HU 2018 graduating class medical students, who agreed to answer the questionnaire. Two-hundred ten medical interns were included to the survey based on a single population formula. On-hundred five medical interns were selected based on simple the random technique from each university.

### 2.2. Study Design and Period

A cross-sectional survey was employed. The survey was conducted from October 01, 2018, to October 30, 2018.

### 2.3. Survey Tools

ECGs were selected from the ECG textbooks and ECG web blogs. After the ECG strip selection, the previous questionnaire was modified and included in the final questionnaire. Pretested and reviewed structured self-administered questionnaires were distributed to the survey participants. The examination consisted of basic information of GMSs and ECGs, including a specific focus on basic, arrest ECG rhythms, and common emergency ECG abnormalities strips. The ECG examination was administered to GMSs.

### 2.4. Data Analysis

The collected data were coded and entered to SPSS 25. Categorical variables were described as counts and percentages. Continuous variables were described as means, standard deviations, and standard errors. The Mann–Whitney *U* test was used to compare the mean score of groups for their correct answers. The chi-squared test was used to compare differences in ECG interpretation skills, with significance level set at *P<*0.05.

Binary logistic regression was implemented to assess which factors significantly influence competency in ECG interpretation. The outcome variable was the correct answer to at least 14 questions, which is consistent with competency greater than 80%; this is a commonly used threshold for a good grade on exams [3].

### 2.5. Inclusion and Exclusion Criteria

#### 2.5.1. Inclusion Criteria

All medical students graduating in 2018 from HU and AAU, who practiced for more than 9 months, were included.

#### 2.5.2. Exclusion Criteria

The exclusion criteria are as follows:

- Who are not willing or unable to participate for different reasons
- On attachment rotation less than 9 months because of possible lack of enough experience and awareness on EM training

### 2.6. Ethical Consideration

An official formal letter was written from Addis Ababa University College of health sciences, Department of Emergency and Critical Care Medicine to get permission to conduct this survey. Involvement of the participants in the survey was on a voluntary basis after getting oral consent. Confidentiality of information was maintained by removing the student's name.

## 3. Results

### 3.1. Characteristics of Survey Participant

In this survey, 202 graduating medical students from both HU (102 GMSs) and AAU (100 GMSs) medical schools participated and completed the self-administered questionnaires. In this survey, the nonresponse rate was 3.8%. Characteristics of the survey participants are displayed in Table 1. Only eighty-two (40.6%) of GMSs from the participant had received ECG training from medical staff other than formal medical school teaching, and 64.6% of GMSs of them were from HU.

One-hundred nineteen (58.9%) of the participants had tried to teach themselves ECG interpretation. Of these, 33.6% use Internet, 30.3% use textbooks, and 21% of GMSs use videos as self-teaching methods.

### 3.2. Competency in ECG Interpretation

The percentage of accurate answers given for each of the 17 ECGs for both AAU and HU GMSs is presented in Table 2. AAU GMSs obtained a significantly higher mean score of answer 8.97 (SE = 0.41) when compared with HU GMSs 3.33 (SE = 0.352) on the 17 ECG strips. In this survey, the overall average of ECG interpretation was 35.9% (95% CI, 32.06–39.8%). The AAU group gained an overall average of 52.65% (95% CI 47.88–57.41%), while the HU group had 19.41% (95% CI 15.47–23.71%) with *P<*0.001. Overall, in this survey, the competency in ECG interpretation was 20.8% (score ≥80%). The competency in ECG interpretations of GMSs of AAU and HU who scored ≥80% was 36.0% and 5.9%, respectively.

#### 3.2.1. Basic ECG Findings

In this survey, 61.3% (95% CI 56.3–66.3%) of GMSs was able to correctly interpret the primary ECG parameters, such as heart rate, heart rhythm, and an electrical axis of the heart (Table 2). Overall, only 33.7% of GMSs was competent (>80%) for basic ECG interpretation. Thus, three basic ECG interpretation mean scores for correct answer were 1.84; SE = 0.079. AAU GMSs had higher mean correct answer than HU GMSs (2.23; SE = 0.079 vs. 1.46; SE = 0.122). The difference was statistically significant with those of the Mann–Whitney *U* test (*U* = 1.84, *P<*0.001).

#### 3.2.2. Arrest Rhythm ECG Abnormalities

The competency (≥80%) of ECG interpretation was assessed for arrest rhythms, like asystole, PEA, Vtac, and Vfib; and only 3 (1.5%) of GMSs were competent, and it is presented in Table 2. There was no GMSs from HU who answered more than 80%. In this survey, the average score of arrest rhythm was 32.75% (95% CI 28.25–37.25). AAU GMSs had higher mean correct answer than HU GMSs (2.21; *SE* = 0.016 vs. 0.43; *SE* = 0.081). The difference was statistically significant with that of the Mann–Whitney *U* test (*U* = 1.31, *P<*0.001).

#### 3.2.3. Common Life-Threatening Emergency ECG Abnormalities

Competency of ECG abnormalities for the list 10 ECG strips was assessed, i.e., atrial fibrillation, anterioseptal and inferior ST segment elevation myocardial infraction (STEMI), hyperkalemia, first-degree AV block, second-degree AV block, third-degree AV block, LBBB, LVH, and pericarditis (Table 2).

In this survey, 30.4% (95% CI 26.0–34.9%) of GMSs were able to correctly interpret common life-threatening ECG abnormalities. Overall, only 19.3% of GMSs were competent for interpretation of common life-threatening ECG abnormalities.

### 3.3. Factors Affecting Competency in ECG Interpretation

In the univariable analysis, ECG class, undergraduate emergency medicine rotations, ECG interpretation as part of the exam, university of undergraduate survey, confidence level, and frequency of ECG interpretation per month were associated with the competency of ECG interpretation. In multivariable analysis, place of the university, ECG class, undergraduate emergency medicine rotations, and confidence level were found to have an association with competence in ECG interpretation (Table 3). Since GMSs from AAU have had ECG class and emergency medicine rotation, they were 9.078 times higher in ECG interpretation than HU GMSs (AOR = 9.078 (2.556–32.249)).

## 4. Discussion

Interpretation of ECG abnormalities can be difficult. But, the ability to interpret ECG abnormalities remains a core clinical competency for GMSs and as a physician. Undergraduates do not consistently receive teaching on ECG interpretation, and this can impact confidence [4, 10, 11]. This survey also showed only GMSs from AAU have taken the ECG class.

The overall average of ECG interpretation was 35.9% (95% confidence interval (CI) 32.06–39.8%). Overall, in this survey, the competency in ECG interpretation was 20.8% (score ≥80%). The competency in ECG interpretations of GMSs of AAU and HU, who scored ≥80% were 36.0% and 5.9%, respectively. Likewise, Jablonover et al. found 37% accuracy in ECG interpretation among 231 GMSs [12].

In contrast to Kopeć et al. where most students of clinical years (86%), this survey showed lower (only two third of GMSs) ability of correctly interpret the primary ECG parameters. This survey revealed that the accuracy of ECG interpretation for arrest rhythm was lower than that in the survey done by Kopeć et al. [3]. This difference could be explained by only half of group participants took ECG class and undergraduate emergency medicine rotation.

Similar to this survey, the studies of Mahler et al. described the importance of formal ECG class which achieved a higher score than self-directed survey [13]. Against to this survey, a survey by Kopeć et al. revealed that competency in ECG interpretation was higher in students who reported ECG self-learning methods. This difference is the number of GMSs using self-learning methods was small. Likewise, McAloon et al. studied that there was a direct correlation between the confidence of GMSs and their competency to accurately identify abnormal ECG tracings.

Competency of ECG interpretation of GMSs who took undergraduate emergency medicine rotation and undergraduate ECG class was nine times more than GMSs who did not take emergency medicine rotation. But, there was no association with attending all classes, so assessment methods or teaching approaches should be assessed and changed. Similarly, a study performed at Worcester Royal Hospital on the undergraduate and postgraduate clinical training has demonstrated ECGs are interpreted suboptimal, and competency of GMSs who attended formal teaching program was significantly higher than GMSs who use self-learning methods [10].

There are several strengths to this survey. The respondents were enrolled from both medical schools, who have undergraduate emergency medicine curriculum (AAU) and who did not include HU. The simple randomized method was used to avoid bias. This survey specified the areas of life-threatening and common emergency ECG abnormalities interpretation skills that need to be improved. It clearly revealed the factors of competency of ECG interpretation. Finally, this survey reported the impact of undergraduate emergency medicine rotation as a curriculum and current ECG education in medical schools on competency in ECG interpretation.

The limitation of this survey is that ECG interpretation skill of GMSs was not assessed directly on ECG strips on the bedside. There was no nationalized curriculum in Ethiopia which assesses competency in ECG interpretation for GMSs.

## 5. Conclusions

This survey showed GMSs demonstrated low competency in ECG interpretations. Competency of ECG interpretations was significantly improved by undergraduate emergency medicine rotation and undergraduate ECG class. Unfortunately, attending all classes was not associated with competency of the ECG interpretation skill. Moreover, most students reported that the number of ECG classes during medical education was insufficient. This shows different teaching models should be applied for ECG interpretations.

## 6. Recommendation

Based on this survey finding, undergraduate emergency medicine rotation and formal ECG class should be included in a curriculum to all medical schools in Ethiopia, as this improves the accuracy of diagnosis and patient outcome. Amount of ECG class should be extended, and teaching methods should be revised and changed.

Further large study should be done on the assessment of competency of ECG interpretations among GMSs.

## Figures and Tables

**Table 1:** Characteristics of the survey participant.

		Place		Total
		AAU	HU	
Age (years) mean ± SD		24.1 (±0.689)	25.21 (±1.037)	24.66 ± 1.04

Gender	Male	55%	60.8%	57.9%
	Female	45%	40 39.2%	42.1%
Received ECG class		100%	—	49.5%

Fully attending ECG class	Yes	85%	—	
	No	15%	—	

Amount of ECG classes	Enough	22%	—	
	Not enough	78%	—	

ECG interpretation as part of the exam	Yes	72%	—	35.6%
	No	28%	—	13.9%

Frequency of ECG machine use per month	<5	22%	21.6%	21.8%
	5–10	6%	5.9%	5.9%
	>10	30%	12.7%	21.3%
	Not at all	42%	59.8%	51.0%

Frequency of ECG interpretations per month	<5	49.0%	37.2%	43.1%
	5–10	6.0%	2.0%	4.0%
	>10	15.0%	1.0%	7.9%
	Not at all	30.0%	59.8%	45.0%

Asking help for ECG interpretations	Rarely	6%	2.9%	4.5%
	Sometimes	36%	27.5%	31.7%
	Always	52%	65.7%	58.9%
	Not at all	6%	3.9%	5.0%

Self-confidence rating for ECG interpretations	Confident	12%	9.8%	10.9%
	Neutral	42%	35.3%	38.1%
	Not confident	46%	54.9%	51%

Consulted physician for ECG abnormalities	Medical intern	7%	2%	4.5%
	GP	2%	5.9%	4%
	Resident	70%	63.7%	66.8%
	Internist	10%	28.4%	19.3%
	Emergency physician	11%	—	5.4%

**Table 2:** Correct answer for each ECG abnormalities.

ECG finding	Place		Total (%)	Pearson chi-squared test
	AAU (%)	HU (%)		
Heart rate	85.0	47.1	65.8	<0.001
Rhythm	91.0	62.7	76.7	<0.001
Axis	46.0	38.2	42.1	0.165
Asystole	73.0	19.6	43.1	<0.001
Ventricular tachycardia	72.0	10.8	41.1	<0.001
Ventricular fibrillation	70.0	12.7	41.1	<0.001
PEA	5.0	—	2.5	0.028
Atrial fibrillation	56.0	22.5	39.1	<0.001
STEMI (anteroseptal)	71.0	14.7	42.6	<0.001
STEMI (inferior)	41.0	12.7	26.7	<0.001
Hyperkalemia	45.0	21.6	33.2	<0.001
First degree AV block	56.0	18.6	37.1	<0.001
Second degree AV block	45.0	5.9	25.2	<0.001
Third degree AV block	38.0	10.8	24.3	<0.001
LBBB	42.0	8.8	26.7	<0.001
LVH	21.0	12.7	16.8	0.044
Pericarditis	50.0	16.7	33.2	<0.001

PEA: pulseless electrical activity; STEMI: ST segment elevation myocardial infraction; AV: atrioventricular; LBBB: left bundle branch block; LVH: left ventricular hypertrophy.

**Table 3:** Factors associated with competency of ECG interpretation.

Factors		ECG Interpretation		COR (95% CI)	AOR (95% CI)
		<8%	≥80%
Gender	Male	77.8	2.2	1.232 (0.614–2.474)	
	Female	81.2	18.8	1	
ECG class	Yes	64.0	36.0	9.000 (3.586–22.591)^*∗*^	9.078 (2.556–32.249)^*∗*^
	No	94.1	5.9	1	1
Place	AAU	64	36.0	9.000 (3.586–22.591)^*∗*^	9.078 (2.556–32.249)^*∗*^
	HU	94.1	5.9	1	1
Attending all classes	Yes	60.0	40.0	3.923 (0.826–18.634)	
	No	86.7	13.3	1	
Undergraduate emergency medicine rotations	Yes	64.0	36.0	9.000 (3.586–22.591)^*∗*^	^*∗*^
	No	94.1	5.9	1	1
ECG interpretation as part of exam	Yes	61.1	38.9	5.273 (2.543–10.935)^*∗*^	1.031 (0.363–2.932)
	No	81.7	18.3	1	1
ECG training	Yes	77.0	23.0	1.437 (0.725–2.848)	
	No	82.0	18.0	1	
Confidence level	Confident	72.7	27.3	3.875 (1.213–12.376)^*∗*^	3.983(1.053–15.064)^*∗*^
	Neutral	65.4	34.6	5.471 (2.39–12.522)^*∗*^	6.057 (2.362–15.533)^*∗*^
	Not confident	91.2	8.8	1	1
Frequency of ECG machine use per month	<5	81.8	18.2	1.065 (0.471–2.411)	
	5–10	63.6	36.4	2.353 (0.635–8.715)	
	>10	67.9	32.1	2.229 (0.862–5.765)	
	Not at all	82.5	17.5	1	
Frequency of ECG interpretation per month	<5	74.7	25.3	1.924 (0.870–4.256)	0.901 (0.349–2.326)
	5–10	62.5	37.5	3.950 (0.834–18.701)	1.217 (0.209–7.084)
	>10	68.8	31.2	4.788 (1.602–14.306)^*∗*^	1.075 (0.266–4.348)
	Not at all	86.8	13.2	1	
Ask for help for interpretation of ECG	Rarely	66.7	33.3	2.000 (0.250–15.991)	
	Sometimes	82.8	17.2	0.830 (0.155–4.455)	
	Always	78.2	21.8	1.118 (0.224–5.559)	
	Not at all	80.0	20.0	1	
Self-learning methods	Yes	77.0	23.0	1.364 (0.680–2.375)	
	No	83.0	18.0	1	

COR: reported crude odds ratios; AOR: adjusted odds ratio *P*: *P* value of test statistic; CI: confidence interval; ^*∗*^statistically significant variable.

## Data Availability

The data used to support the findings of this study are available from the corresponding author upon request.
